# Dr. Walter Wyman

**Published:** 1911-12

**Authors:** 


DR. WALTER WYMAN, Surgeon General of the same Service died at Providence Hospital, Washington, November 21, 1911. We feel a sense of personal loss in the death of these scholarly gentlemen. The writer was appointed to the service in 1900 but did not serve, on account of personal and business reasons. However, the acquaintance formed through this interest in the service has lasted over many years, with frequent meetings.

